# Edaravone, a Free Radical Scavenger, Delayed Symptomatic and Pathological Progression of Motor Neuron Disease in the Wobbler Mouse

**DOI:** 10.1371/journal.pone.0140316

**Published:** 2015-10-15

**Authors:** Ken Ikeda, Yasuo Iwasaki

**Affiliations:** Department of Neurology, Toho University Omori Medical Center, Tokyo, Japan; University of Sheffield, UNITED KINGDOM

## Abstract

Edaravone, a free radical scavenger is used widely in Japanese patients with acute cerebral infarction. This antioxidant could have therapeutic potentials for other neurological diseases. Amyotrophic lateral sclerosis (ALS) is a fatal neurodegenerative disease that affects the upper and the lower motor neuron, leading to death within 3–5 years after onset. A phase III clinical trial of edaravone suggested no significant effects in ALS patients. However, recent 2nd double-blind trial has demonstrated therapeutic benefits of edaravone in definite patients diagnosed by revised El Escorial diagnostic criteria of ALS. Two previous studies showed that edaravone attenuated motor symptoms or motor neuron degeneration in mutant s*uperoxide dismutase 1*-transgenic mice or rats, animal models of familial ALS. Herein we examined whether this radical scavenger can retard progression of motor dysfunction and neuropathological changes in wobbler mice, sporadic ALS-like model. After diagnosis of the disease onset at the postnatal age of 3–4 weeks, wobbler mice received edaravone (1 or 10 mg/kg, n = 10/group) or vehicle (n = 10), daily for 4 weeks by intraperitoneal administration. Motor symptoms and neuropathological changes were compared among three groups. Higher dose (10 mg/kg) of edaravone treatment significantly attenuated muscle weakness and contracture in the forelimbs, and suppressed denervation atrophy in the biceps muscle and degeneration in the cervical motor neurons compared to vehicle. Previous and the present studies indicated neuroprotective effects of edaravone in three rodent ALS-like models. This drug seems to be worth performing the clinical trial in ALS patients in the United States of American and Europe, in addition to Japan.

## Introduction

Edaravone is a free radical scavenger, and it has been used in numerous patients with acute cerebral infarction in Japan from 2001. Edaravone has also been introduced in the United States of America (USA) for the early management of adults with ischemic stroke [1–3]. The antioxidant mechanism of edaravone seems to increase prostacyclin production, decrease lipoxygenase metabolism of arachidonic acid by trapping hydroxyl radicals, and inhibit alloxan-induced lipid peroxidation and quench active oxygen. Moreover, these protective effects can act on various kinds of cells, including neurons, endothelial cells and myocardial cells, against damage by reactive oxygen species [4].

Amyotrophic lateral sclerosis (ALS) is a devastating neuromuscular disorder characterized by progressive degeneration of the upper and the lower motor neuron. Patients suffer from atrophy and paralysis of the bulbar, the limb and the respiratory muscle, leading to death at 3–5 years after disease onset. In addition to ischemic stroke, edaravone has therapeutic potentials on several neurological diseases [4, 5]. Especially, recent 2nd clinical trial of edaravone has provided benefits in definite ALS patients according to revised El Escorial diagnostic criteria whereas there were no significant effects in the first phase III trial [6]. Two previous studies using rodent models of familial ALS reported disease-slowing effects by edaravone treatment immediately after disease onset or from pre-onset. Wobbler mouse revealed neuromuscular deficits and neuropathological findings partially mimicking sporadic ALS [7–10]. We previously examined therapeutic responses of several agents in this mouse [11–18]. The present study was aimed to whether symptomatic and neuropathological changes can be ameliorated in wobbler mice treated with edaravone after onset of motor neuron disease.

## Materials and Methods

### Animals

The original wobbler mouse mutation (wr) occurred spontaneously as an autosomal recessive mutation in the congenic C57BL/6J mouse strain [7]. The original colony was crossed with a high fertility strain (NFR/N) and maintained. Wobbler mice (wr / wr) and their normal littermates (wr / + or + / +) in this study were raised in the Fourth Department of Internal Medicine, Toho University mouse colony, Tokyo, Japan. The present study was carried out in strict accordance with the recommendations in the Guide for the Care and Use of Laboratory Animals of Toho University. The protocol was approved by the Committee on the Ethics of Animal Experiments of Toho University Ohashi Hospital.

### Randomized blind administration of edaravone

Edaravone was supplied from Mitsubishi Tanabe Pharma Corporation, Tokyo, Japan. For the randomized control study, we arbitrarily determined to analyze 10 animals per group, in the similar way to previous studies [11–18]. Each mouse was assigned confidentially in numerical order into one of 3 different doses of edaravone groups (0 mg/kg, 1 mg/kg, and 10 mg/kg). This automatic procedure of randomization thoroughly eliminated the controller's bias, and it was difficult to conceive the dose of edaravone for each mouse by its number. The researchers who participated in this study were strictly blind to the information of treatment. The wobbler mice initially developed shaking body from the age of 3 to 4 weeks, and were diagnosed as the symptomatic onset. Immediately after diagnosis, edaravone or vehicle was administered by intraperitoneal injection daily for 4 weeks. Finally, participating affected mice were divided into three groups: the low dose edaravone group (1 mg/kg, n = 10), the high dose edaravone group (10 mg/kg, n = 10) and the vehicle group (n = 10). The treatment finished at the age of 7 to 8 weeks.

### Symptomatic assessment

Forelimb deformity was graded as 1, paw atrophy; 2, curled digits; 3, curled wrists; and 4, forelimb flexion to chest. Pull strength of both forelimbs was measured as described previously [11–18]. These assessments were performed weekly from the beginning (pre-treatment) to the end of treatment. The physical condition of all mice was monitored daily by the experimental researcher (KI) and two animal stuffs. Wobbler mice had no hindlimb deformity during this study. Mice experienced no difficulty with locomotion or with accessing food/water. There were no dead or euthanized mice prior to the experimental endpoint of the present study.

### Morphometry of biceps muscle

After treatment, wobbler mice (n = 10/group) were anesthetized with intraperitoneal injection of pentobarbital sodium (40 mg/kg), and the right biceps muscles were removed under a dissecting microscope. They were accurately weighed and frozen. Serial 10 μm sections were stained with routine ATPase. The mean diameter of muscle fibers was determined using morphometric system, MacScope^®^ (Mitani Corporation, Fukui, Japan). Morphometric analysis was also done in five age-matched healthy littermates (3 male and 2 female mice) for comparison as the wild type control.

### Number of spinal motor neurons

Wobbler mice (n = 10/group) that were used for morphometric analysis of the biceps muscle were perfused through an intracardiac catheter with phosphate buffered saline followed by 4% paraformaldehyde/1% glutaraldehyde/0.1 M sodium phosphate buffer, pH 7.4. Laminectomy was performed, and the cervical spinal cord was removed under a dissecting microscope. C5-6 segments, which innervate the biceps muscles, were taken for analysis of motor neurons. The spinal cord segments were embedded in paraffin, sectioned serially at 8 μm in the transverse plain, and stained with cresyl-violet. The number of motor neurons was determined by the previous method [11–18]. That was also counted in five normal littermates used for the morphometry of the biceps muscle.

### Density of astrocytes in cervical cord

For immunohistochemistry of glial fibrillary acidic protein (GFAP) to identify astrocytes, paraffin-embedded sections used for spinal motor neuron counting were deparaffinized and incubated overnight at 4°C with rabbit antibody to GFAP (Dako, Denmark) at a dilution of 1:1000. GFAP-immunoreactive astrocytes were investigated with a light microscope at magnification X 200. The number of astrocytes (cells/mm^2^) was measured in the ventral horn of the C5-6 cord, using a computer-associated image analyzer software (MacScope^®^) as previously described [17].

### Statistical analyses

All data are shown as mean ± SEM. The difference between the vehicle group and edaravone groups in the scale of forelimb deformity and grip strength were analyzed by one-way repeated measures ANOVA followed by Dunnett multiple comparison test. Dunnett test was performed between groups at each of the four time points from the 1st to the 4th week. The weight and the mean diameter of biceps muscle, the number of motor neurons, and the density of astrocytes were analyzed using one-way ANOVA followed by Dunnett multiple comparison tests. As the vehicle group was set as the control in the Dunnett test, the two-group comparison was performed between the high dose group and the vehicle group, and between the low dose group and the vehicle group. *P* value of < 0.05 was considered to be significant. Statistical analyses were performed using PASW Statistics 18.0 (IBM, Chicago, the USA).

## Results

### General information of participating wobbler mice

A total of 30 wobbler mice (15 male and 15 female mice) were participated in the present study. Five male and 5 female mice existed in each group. The body weight of mice was 6.5–8.0 g at the starting point of this study. The body weight was measured daily from the beginning to the end of treatment. The weight gain did not differ among three groups. The body weight was increased to 7.5–9.5 g at the final treatment day.

### Motor symptoms in the forelimbs

**Fig 1:**
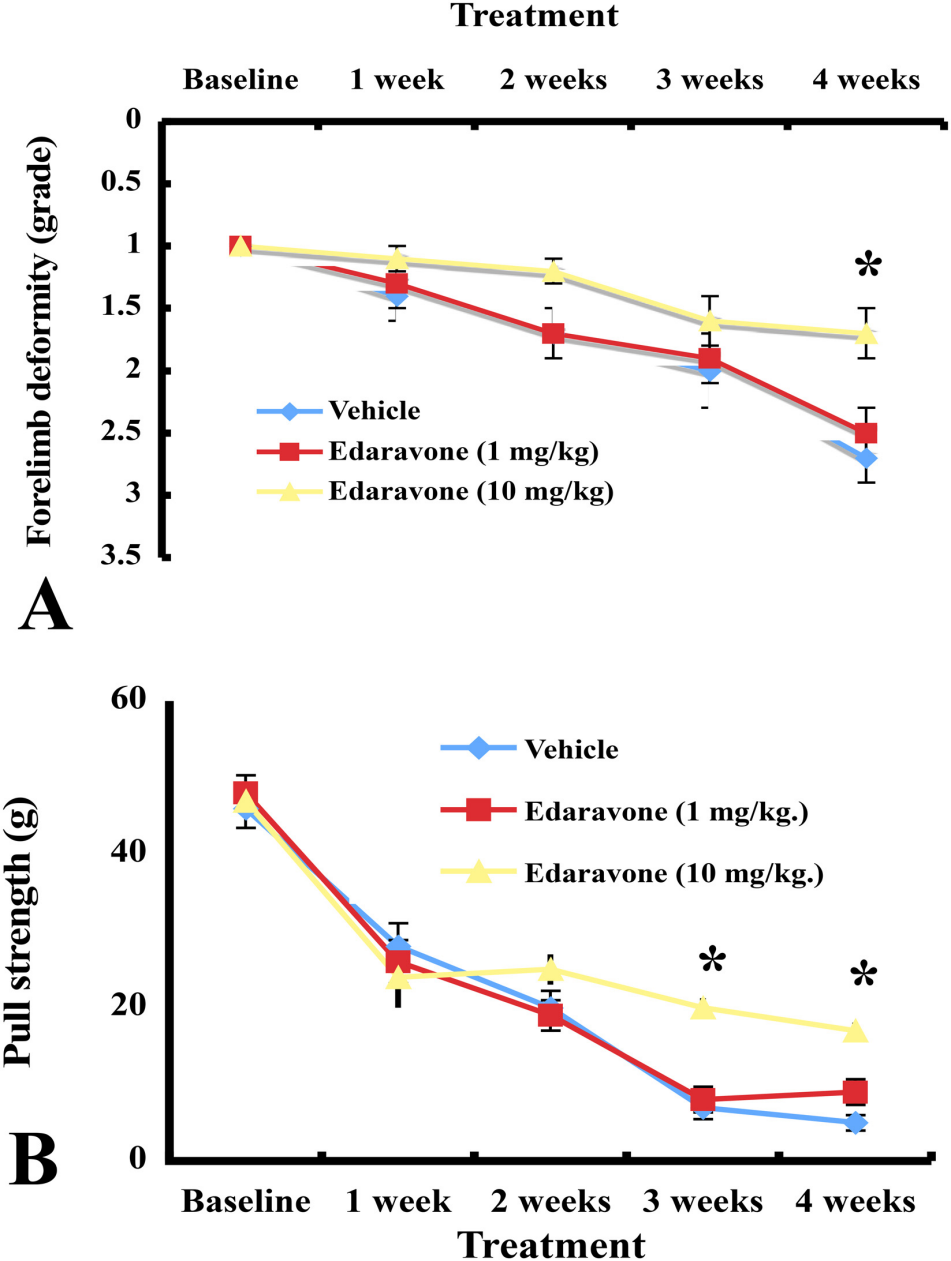
Forelimb deformity and pull strength changes after treatment with edaravone and vehicle. (A) The forelimb deformity was attenuated in mice treated with higher dose of edaravone. * *P*<0.05, the higher dose of edaravone group versus the vehicle group at the 4th week (one-way repeated measures ANOVA followed by Dunnett type multiple comparison test). Results are represented as the mean ± SEM (n = 10/group). (B) Vehicle-treated mice decreased forelimb strength gradually. Higher dose of edaravone administration inhibited deterioration of forelimbs strength. * *P*<0.05, the higher dose of edaravone group versus the vehicle group at the 3rd and the 4th week (one-way repeated measures ANOVA followed by Dunnett type multiple comparison test). Results are represented as the mean ± SEM (n = 10/group).

The assessments at baseline (postnatal age of 3–4 weeks) did not differ among three groups. Forelimb deformity deteriorated progressively in vehicle-treated mice. The progression of forelimb deformity was suppressed significantly at 4 weeks after treatment with higher dose of edaravone compared to vehicle (*P< 0*.*05*). Forelimb deformity score did not differ statistically between the lower dose group and the vehicle group (Fig 1A). Forelimb pull strength was reduced gradually in vehicle-treated wobbler mice. As compared to vehicle, mice treated with higher dose edaravone attenuated muscle weakness in the forelimbs after 3 weeks (*P< 0*.*05*) and 4 weeks (*P< 0*.*05*). Forelimb strength did not differ significantly between lower dose edaravone and vehicle treatment (Fig 1B).

### Biceps muscle weight and morphometry

**Fig 2:**
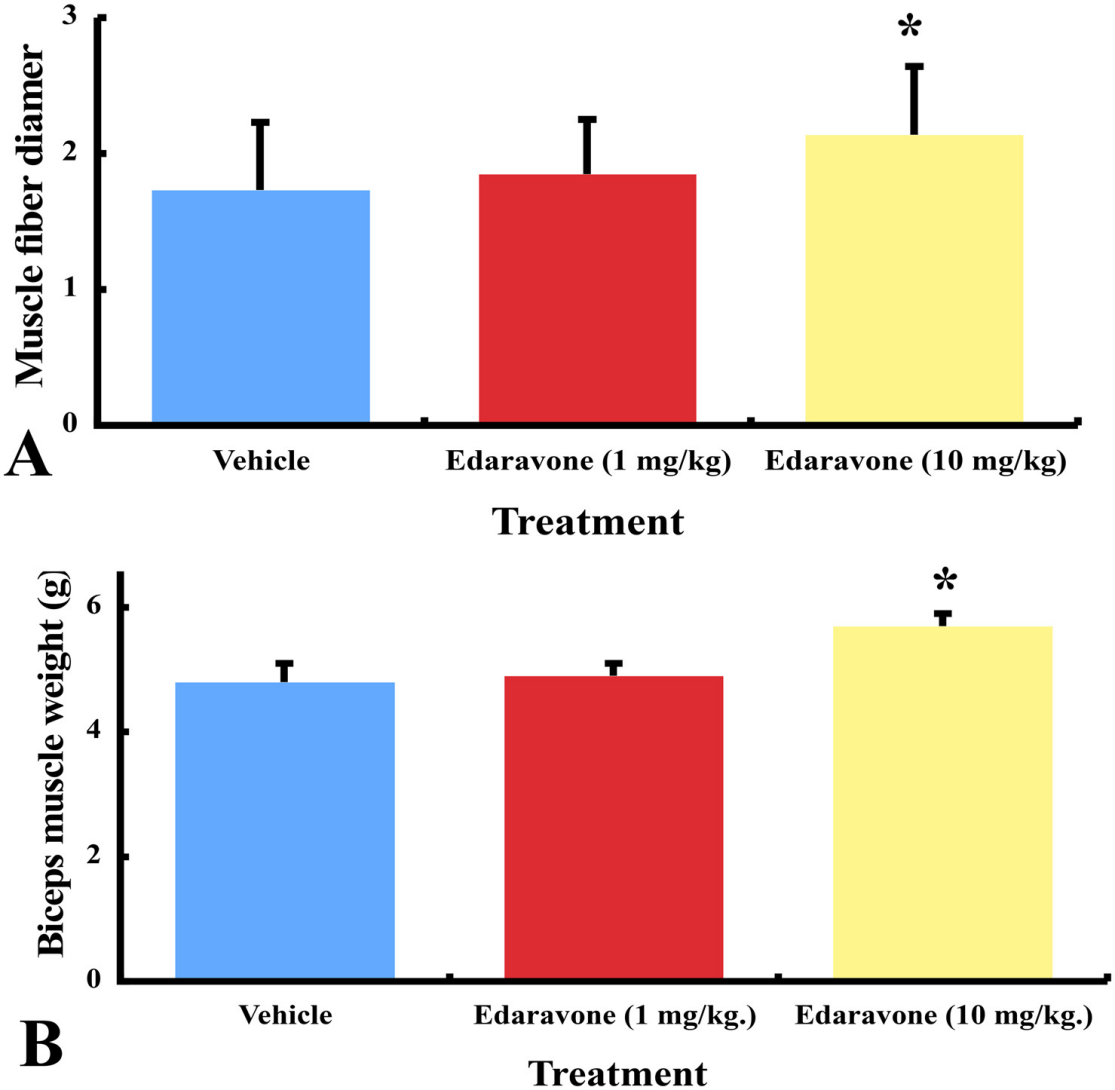
The biceps muscle weight and morphometry in edaravone- and vehicle-treated wobbler mice. (A) Mice treated with higher dose of edaravone increased significantly the weight of biceps muscles compared to vehicle. * *P*<0.05, the higher dose of edaravone group versus the vehicle group (one-way ANOVA followed by Dunnett multiple comparison test). Results are represented as the mean ± SEM (n = 10/group). (B) Mice treated with higher dose of edaravone increased the diameter of muscle fibers significantly compared to vehicle. * *P*<0.05, the higher dose of edaravone group versus the vehicle group (one-way ANOVA and Dunnett multiple comparison test). Results are represented as the mean ± SEM (n = 10/group).

The mean weight ± SEM of the biceps muscle was 4.8 ± 0.3 mg in the vehicle group, 4.9 ± 0.2 in the lower dose edaravone group, and 5.7 ± 0.2 in the higher dose edaravone group. The biceps muscle weight was significantly increased in the higher dose edaravone group, compared to the vehicle (*P< 0*.*05*) [Fig 2A].

The mean diameter ± SEM of muscle fibers was 21.4 ± 0.5 μm in the higher edaravone dose group, 18.5 ± 0.4 in the lower dose edaravone group, and 17.3 ± 0.5 in the vehicle group (Fig 2B). Higher dose edaravone treatment increased the diameter of muscle fibers significantly compared to vehicle (*P< 0*.*05*). In normal littermates, the mean weight and the mean fiber diameter of the biceps muscle were 15.5 ± 1.0 mg and 41.9 ± 1.2 μm, respectively.

### Survival of spinal motor neurons

**Fig 3:**
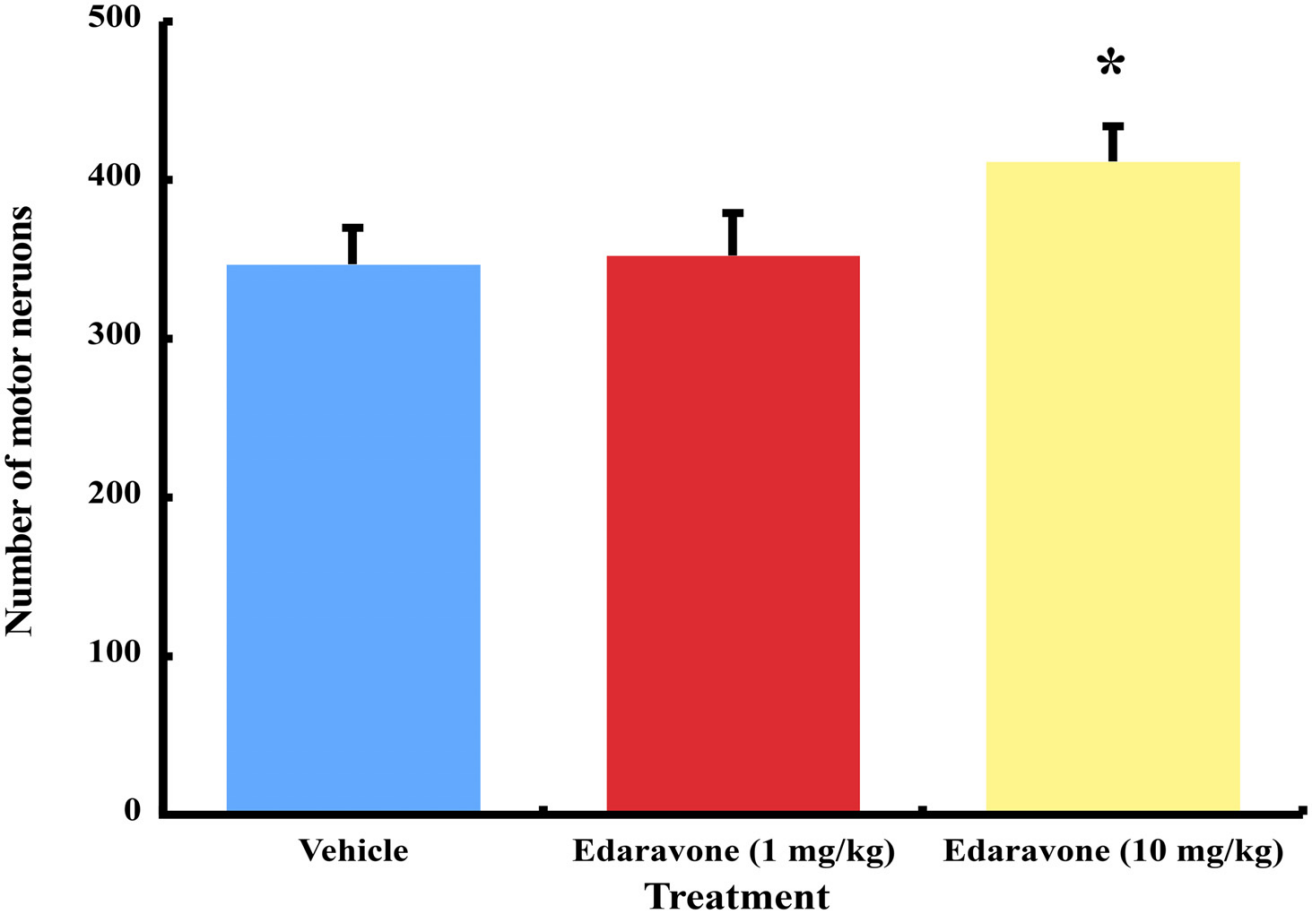
The number of spinal motor neurons in edaravone- and vehicle-treated wobbler mice. Mice treated with higher dose of edaravone increased the number of motor neurons significantly compared to vehicle. * *P*<0.05, the higher dose of edaravone group versus the vehicle group by one-way ANOVA and Dunnett multiple comparison test. Results are represented as the mean ± SEM (n = 10/group).

The mean number ± SEM of spinal motor neurons was 346.7 ± 23.3 in the vehicle group, 352.4 ± 26.9 in the lower dose edaravone group, and 411.6 ± 22.2 in the higher dose edaravone group. The number of motor neurons differed significantly between wobbler mice treated with the higher dose of edaravone and vehicle (*P<0*.*05*). The statistical significance was not found between the lower dose edaravone group and the vehicle group (Fig 3). The mean number ± SEM of spinal motor neurons was 1101.7 ± 122.1 in normal littermates.

### Astrocyte proliferation

**Fig 4:**
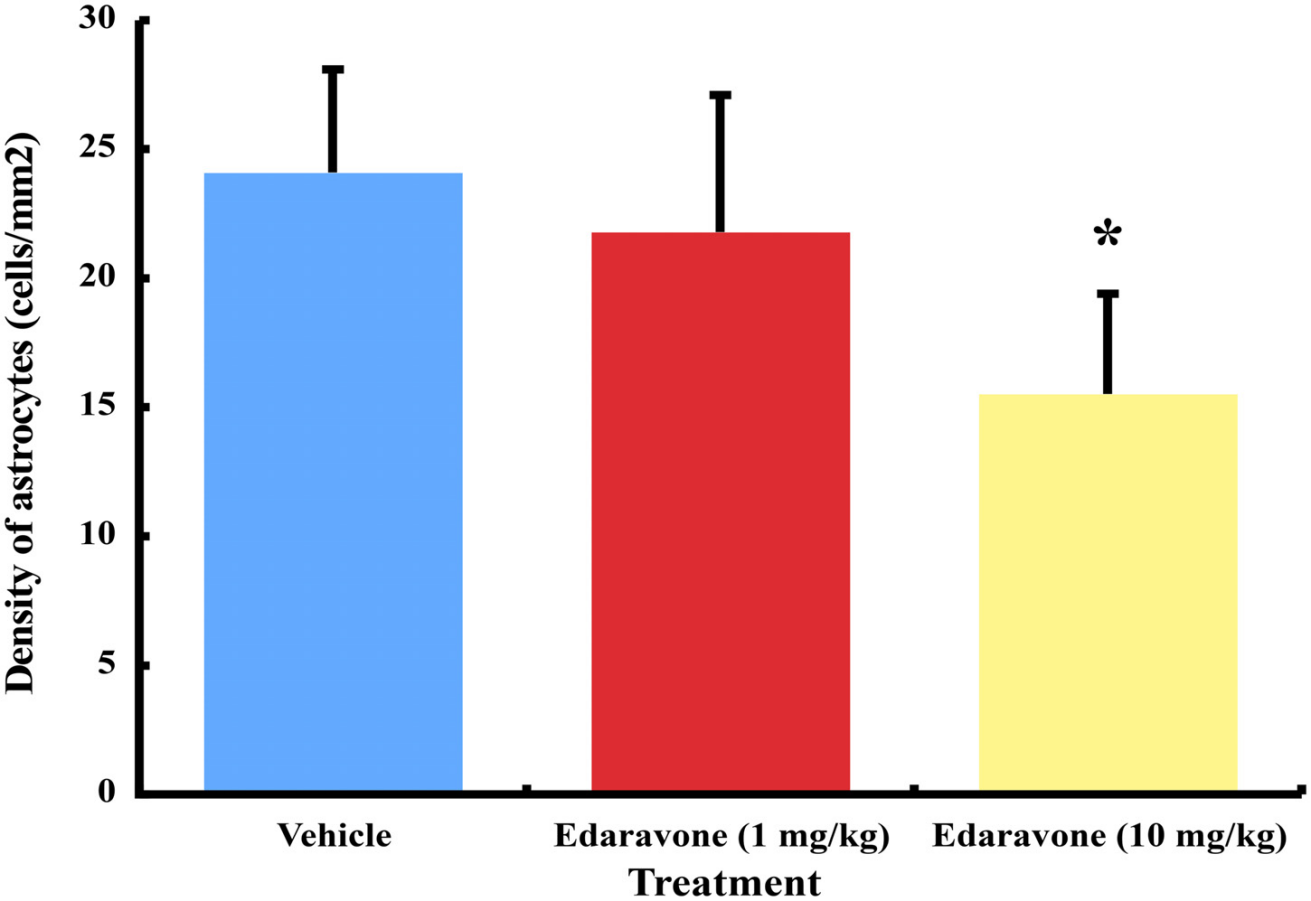
The astrocyte density of the ventral spinal cord in edaravone- and vehicle-treated wobbler mice. Mice treated with higher dose of edaravone decreased the density of GFAP-immunoreactive astrocytes significantly compared to vehicle. * *P*<0.05, the higher dose of edaravone group versus the vehicle group by one-way ANOVA and Dunnett multiple comparison test. Results are represented as the mean ± SEM (n = 10/group).

The density of GFAP-immunoreactive astrocytes was 15.5 ± 3.9 cells/mm^2^ in higher dose of edaravone, 21.8 ± 5.3 in lower dose of edaravone and 24.1 ± 4.0 in vehicle (Fig 4). Higher dose of edaravone treatment inhibited the proliferation of astrocytes significantly compared to vehicle (*P<0*.*05*).

## Discussion

Wobbler mice exhibit autosomal recessive traits of disease and trembling of the body occurs from the age of 3 to 4 weeks. Muscle weakness and deformity in the forelimbs progress rapidly until 7–8 weeks of age. Thereafter, these neuromuscular deficits progress slowly [8]. The pathological hallmarks are spinal motor neuron loss and proximal axonopathy predominantly in the cervical cord, leading to denervation muscle atrophy in the forelimb [9]. Therefore, these clinico-pathological changes from the age of 3–4 weeks to 7–8 weeks show that the wobbler mouse is a useful animal model for evaluating neuroprotective agents [11–18].

Recently, wobbler mice have been shed light upon by their similarities of the molecular pathogenesis to ALS [10]. The causative gene of wobbler mice was found as *Vps54*, which encodes a component of Golgi-associated retrograde protein complex. The latter is involved in intracellular vesicular trafficking and tethers vesicles derived from endosomes to the trans Golgi network [19]. Their human counterpart is not known, but the functions of a number of hereditary ALS genes have been related to vesicular trafficking and Golgi networks (ALS2, ALS8, ALS12, ALS14, and ALS17) [20–24]. Abnormal localization of TDP-43, the pathological hallmark of sporadic ALS was also present in wobbler mouse model [25]. It is therefore relevant to consider the wobbler mouse as a model of sporadic ALS and in fact has long been used as a model to examine possible therapeutic agents for ALS patients [11–18, 26].

In the present study, intraperitoneal administration of higher-dose edaravone (10 mg/kg) attenuated muscle weakness, muscle contracture, denervation muscle atrophy in the forelimb, astrocyte proliferation and motor neuron degeneration of the cervical cord in wobbler mice. These significant effects were not found by treatment with lower dose of edaravone (1 mg/kg). A previous pharmacodynamic study after a single intraperitoneal administration of edaravone described that the daily dose of edaravone might be 15 mg/kg for effective plasma concentrations in mutant *superoxide dismutase (SOD) 1*-transgenice mice, a familial ALS-like model [27]. Two previous studies revealed therapeutic effects of edaravone in such ALS-like rodents. The first study reported that immediate treatment with edaravone (15 mg/kg/day) after disease onset delayed progression of motor symptoms and motor neuron degeneration in mutant *SOD1 (G93A)*-transgenic mice [27]. In another study, continuous infusion of edaravone (3 mg/kg/day) was performed from the pre-symptomatic stage of male mutant *SOD1 (H46R)*-transgenic rats [28]. Those rats received the same dose of edaravone which was administered in ALS patients at Japanese Phase III clinical trial [6]. The landing foot-splay test was improved significantly [28].

The antioxidant mechanism of edaravone seems to increase prostacyclin production, decrease lipoxygenase metabolism of arachidonic acid by trapping hydroxyl radicals, inhibit alloxan-induced lipid peroxidation and quench active oxygen [4]. Edaravone has been found to have other properties besides antioxidant actions. Successful treatment with edaravone was reported in several animal models of subarachnoid hemorrhage, intracerebral hemorrhage, spinal cord injury, traumatic brain injury, radiation brain injury, ALS, Parkinson's disease and multiple sclerosis [4, 5]. In the present study, the precise mechanism of high-dose edaravone remains unknown in wobbler mice. Edaravone has neuroprotective effects on spinal cord neurons by suppressing the level of free radical species in rabbit spinal cord injury [29, 30]. This antioxidant was reported to prevent spinal cord damage by reduction of neuronal nitric oxide synthase (nNOS) levels and potentiation of SOD1 levels after transient ischemia in rabbits [31]. The deposition of abnormal SOD1 in the spinal motor neuron and the ratio of 3-nitrotyrosine/tyrosine were decreased by edaravone administration in mutant *SOD1 (G93A)*-transgenic mice [27]. Our previous studies suggested that nNOS inhibitor or lecithinized SOD treatment attenuated wobbler mouse motor neruon disease [12, 14]. Both antioxidant effects and repairing effects of nNOS and SOD1 activities could contribute to the therapeutic mechanism of edaravone in wobbler and mutant *SOD1*-transgenic mice. With respect to inhibition of astrocytosis in edaravone-treated wobbler mice, the biological mechanism remains unclear in the present study. Astrocyte proliferation occurs together with loss of motor neurons in wobbler mice [17]. Therefore, edaravone treatment might decrease astrocyte proliferation as the result from attenuating motor neuron degeneration. This drug seemed to interact indirectly or directly on astrocytes and motor neurons in wobbler mice.

The limitation of the present study included the short treatment for 4 weeks and small number of animals. Previous treatment studies were performed usually at the number of 10 wobbler mice per group [11–18]. Therefore, we determined the number of 10 mice/group as the minimum number of the therapeutic evaluation. Whether longer treatment with higher dose of edaravone may have more therapeutic benefits is crucial. We determined the therapeutic duration from the onset of postnatal 3–4 weeks to 7–8 weeks. This period was the most active and rapidly progressive stage of motor neuron disease in wobbler mice. The natural disease course revealed that muscle weakness progressed very slowly after the age of 9 weeks. Generally, the survival effect is the most sensitive and reliable symptomatic indicator of treatment. The survival time of wobbler mice is approximately 1.0–1.5 years when normal littermates live as caregivers in the same cage [8]. Without normal littermates, affected mice suffer from eye-opening failure by the accumulation of eye mucus, ocular infection and impossible grooming due to abolition of motor function in both forelimbs. If a wobbler mouse was fed alone in the cage after the symptomatic onset, we experienced that the mouse died frequently within 1–2 months. Therefore, we could not assess adequate survival effects of edaravone because of the ethical problem and the animal property.

## Conclusions

The present experimental study highlighted protective effects of edaravone in wobbler mouse motor neuron disease. A number of agents that had proved to effect in animal models of ALS, including mutant *SOD1*-transgenic mouse, failed in clinical trials of ALS patients [32]. Although this study is a small number analysis, edaravone is the first beneficial drug in both wobbler and mutant *SOD1 (G93A)*-transgenic mice. We expect to perform the clinical trial of edaravone in ALS patients in the USA and Europe, in addition to Japan.
